# Supplementary material for: Viruses of Freshwater Mussels during Mass Mortality Events in Oregon and Washington, USA
Source: Viruses. 2023 Aug 11;15(8):1719. doi: 10.3390/v15081719 (PMC10458741; doi:10.3390/v15081719)
Supplement: Supplementary file 1 [file viruses-15-01719-s001.zip › viruses-2501080-supplementary.pdf]

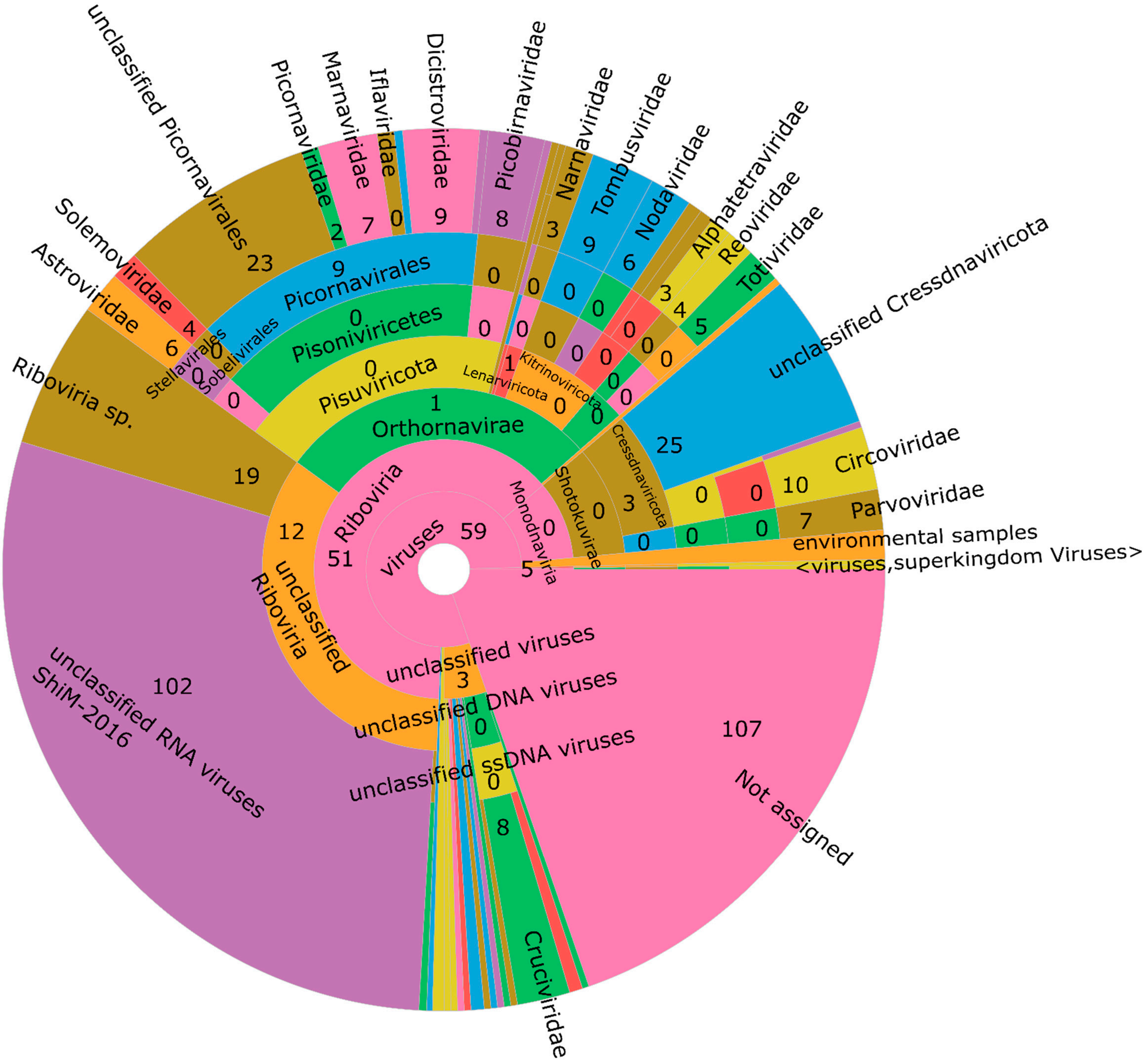

Supplemental Figure S1. Original classifications for all 557 virus contigs of western freshwater mussels assigned by the DIAMOND + MEGAN pipeline. The largest group was "not assigned" (i.e., not even identified as viral taxa) followed closely by uncharacterized viruses described by Shi et al. (2016).
